# Mechanotransduction pathways in the regulation of cartilage chondrocyte homoeostasis

**DOI:** 10.1111/jcmm.15204

**Published:** 2020-04-01

**Authors:** Zhenxing Zhao, Yifei Li, Mengjiao Wang, Sen Zhao, Zhihe Zhao, Jie Fang

**Affiliations:** ^1^ State Key Laboratory of Oral Diseases National Clinical Research Center for Oral Diseases West China Hospital of Stomatology Sichuan University Chengdu China; ^2^ Department of Pediatrics West China Second University Hospital Sichuan University Chengdu China; ^3^ Ministry of Education Key Laboratory of Women and Children's Diseases and Birth Defects West China Second University Hospital Sichuan University Chengdu China; ^4^ Department of Orthodontics School of Dentistry Chonbuk National University Jeonju Korea

**Keywords:** cartilage, chondrocyte, mechanotransduction signalling, osteoarthritis

## Abstract

Mechanical stress plays a critical role in cartilage development and homoeostasis. Chondrocytes are surrounded by a narrow pericellular matrix (PCM), which absorbs dynamic and static forces and transmits them to the chondrocyte surface. Recent studies have demonstrated that molecular components, including perlecan, collagen and hyaluronan, provide distinct physical properties for the PCM and maintain the essential microenvironment of chondrocytes. These physical signals are sensed by receptors and molecules located in the cell membrane, such as Ca^2+^ channels, the primary cilium and integrins, and a series of downstream molecular pathways are involved in mechanotransduction in cartilage. All mechanoreceptors convert outside signals into chemical and biological signals, which then regulate transcription in chondrocytes in response to mechanical stresses. This review highlights recent progress and focuses on the function of the PCM and cell surface molecules in chondrocyte mechanotransduction. Emerging understanding of the cellular and molecular mechanisms that regulate mechanotransduction will provide new insights into osteoarthritis pathogenesis and precision strategies that could be used in its treatment.

## 1 INTRODUCTION

Weight‐bearing joints, such as the knees, hips and temporomandibular joints, are subjected to continuous mechanical loading. The surfaces of diarthrodial joints are protected by cartilage, which mainly contains collagens, proteoglycans (PGs) and water.^1^ The mechanical properties of articular cartilage widely depend on the matrix microstructure, which is structurally and spatially organized to resist compressive loads throughout a range of motion.

**FIGURE 1:**
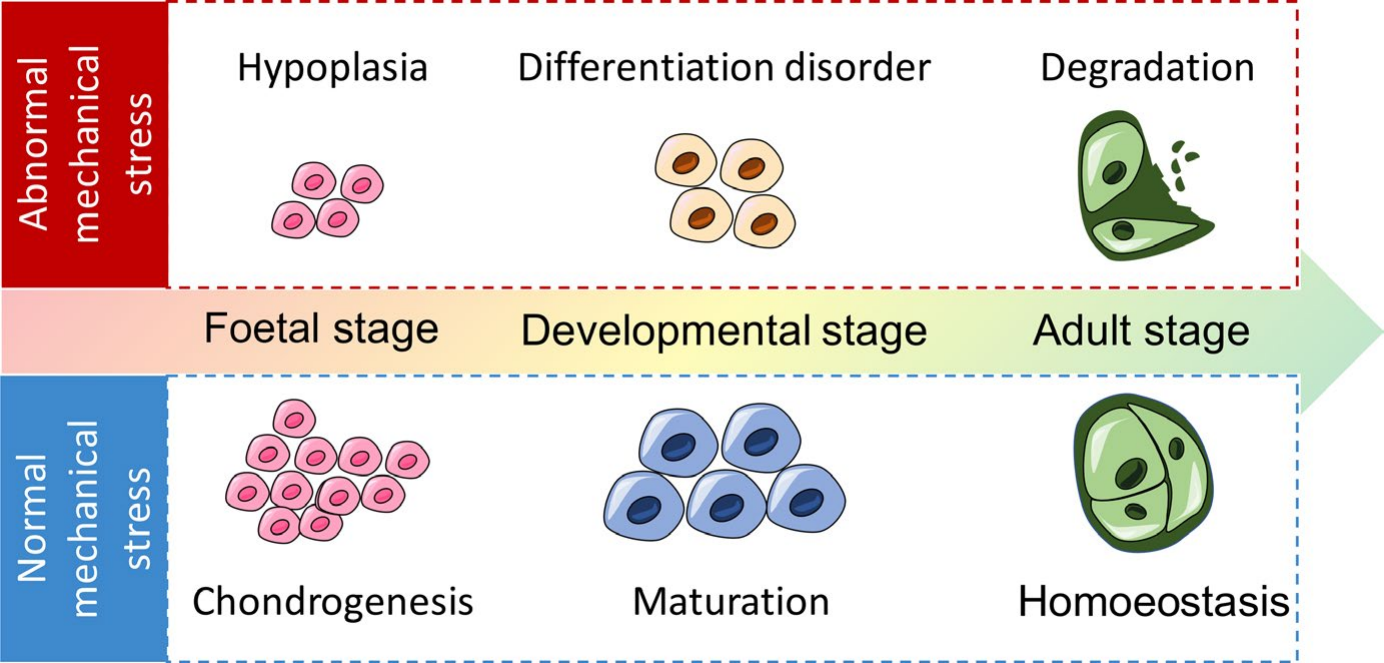
Mechanical stress regulating chondrocyte hemostasis among different stages, while at specific stage, abnormal mechanical stress would induce hypoplasia, differentiated disorder and cartilage degradation

Sensitivity to mechanical signals and adaptive responses to stimuli are essential features of articular cartilage. Chondrocytes, the sole cell type comprising cartilage tissue, experience regular mechanical stimuli during joint loading.^2^, ^3^ The physical microenvironment of chondrocytes has an important impact on cartilage homoeostasis and function.^4^ During normal chondrocyte physiological loading, a balance between anabolic and catabolic processes is reached, manifested as a slow turnover of the cartilage pericellular matrix (PCM), which surrounds the cell. Normally, mechanical stress induces chondrogenesis during foetal development and promotes maturation later in development. In adults, optimal mechanical stress helps chondrocytes maintain stable haemostasis. However, mechanical stress loading from abnormal conditions (eg obesity, joint trauma, instability or malalignment) is occasionally applied to articular cartilage, impairing normal physiological processes. Abnormally applied mechanical stress can induce chondrocyte hypoplasia during foetal development and produce differentiation disorders during subsequent development. According to clinical and animal studies, excessive mechanical stress is instrumental in the initiation and development of osteoarthritis (OA) due to articular cartilage degradation (Figure 1).^5^, ^6^

Previous experimental studies have revealed the importance of mechanotransduction in joint health maintenance as a regulator of transcriptional activity and cartilage homoeostasis. This review highlights emerging advances in understanding of aberrant cellular and molecular mechanisms of mechanotransduction as contributors to OA pathogenesis.

## 2 TRANSCRIPTION CHANGES INDUCED BY PHYSICAL AND MECHANICAL STRESSES

Several types of environmental stimuli can alter specific gene expression, and this process always involves multiple factors working sequentially. Theoretically, chondrocytes are well‐specialized cells that can sense and respond to mechanical stimuli through mechanotransduction processes. A variety of extracellular and pericellular sensors can detect mechanical forces which are then converted into mechanical signals, initiating a biochemical response.

Once a mechanical stress is applied to a joint, the PCM senses environmental changes and deforms as a physical response. A set of molecules located in the PCM are released to target their receptors. Thus, chondrocytes respond to mechanical stress in two ways. One response involves the deformation of the PCM after contacting the surface of chondrocytes and the sensor structures. Another response is due to interactions between released factors and their receptors.

In subsequent stages, sensors and receptors located on chondrocyte cell membranes assist with the transformation of physical stimulation into biochemical signalling, in the form of metabolic changes, inflammatory responses and protein phosphorylation. Therefore, cellular responses to mechanical stress cross the cellular membrane into the cytoplasm, completing the conversion from physical impulse into biochemical patterns.

Finally, biochemical signals translocate into the nucleus and alter transcription. This response involves several pathways that ultimately modulate gene expression, generating changes in cell behaviour. Proteins translated as a result can contribute as effectors and target the PCM, damaging or degrading it. This results in a positive feedback loop that removes protection from chondrocytes and induces OA.

## 3 THE PERICELLULAR MATRIX AS A MECHANOTRANSDUCER

### 3.1 PCM physical structure

In normal chondrogenesis, the PCM along with encapsulated chondrocytes is referred to as a “chondron”.^7^ It is approximately 1‐5 µm thick and rich in macromolecules including perlecan,^8^ aggrecan, hyaluronan and biglycan. The PCM also contains collagen (types IX and VI),^9^ but it is usually defined by of the presence of type VI collagen, which is not found in the extracellular matrix (ECM).^10^ These molecules form the distinct ultrastructure of the PCM, and they are the main components in cell‐matrix interactions during mechanotransduction.^11^ *Col6a1^−/−^* mice that have the depletion of *Col6a1* exhibit significantly lower PCM moduli than wild‐type mice, which was confirmed by another study using micropipette aspiration to determine chondron properties.^12^ This supports the dominant role of type VI collagen in controlling the physical properties of the cartilage PCM. Perlecan, a large heparan sulphate (HS) proteoglycan,^8^ is found exclusively in the PCM of normal articular cartilage. Its combination with type VI collagen exhibits lower elastic moduli (range 0.1‐8 kPa) than peripheral regions that are rich in type VI collagen alone, such as the ECM surroundings (range 0.1‐2 MPa).^13^ The PCM's structural properties allow transduction of mechanical signals from the ECM environment to a series of receptors (including ion channels, integrins and primary cilia) located in the cell membrane, which has a mild elastic modulus. The PCM also provides an enclosed microenvironment in which optimal mechanical stress drives chondrogenesis and maintains homoeostasis.

### 3.2 PCM deformation in response to mechanical stress

**FIGURE 2:**
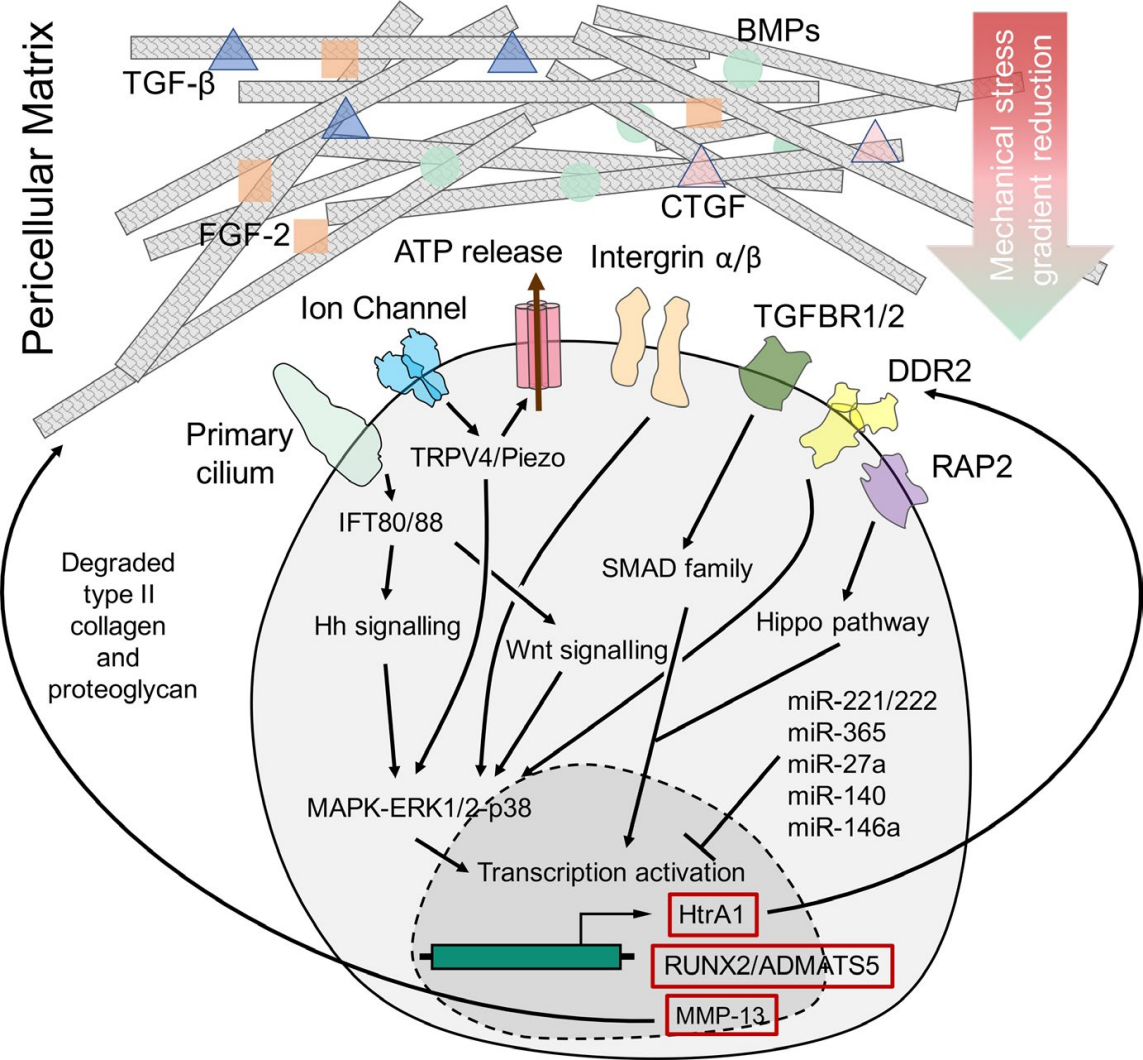
The basis of molecular and intracellular pathways in regulating mechanotransduction signaling. Pericellular matrix firstly regulates mechanical stress and generate a gradient reduction. Several cytokines locates within the pericellular matrix and maintain its biological function. Then the mechanosensor surrounding cell membrane translated physical signaling into chemical and biological signaling, which induce or inhibit specific transcription

The mechanical characteristics of the PCM are distinct from the properties of chondrocytes and the ECM, and the PCM can significantly alter principal stress and strain magnitudes on the surface of chondrocytes.^14^ Theoretical modelling of cell‐matrix interactions indicates that the mechanical environment of chondrocytes is highly heterogeneous, depending on the viscoelastic properties of the PCM.^15^ In situ imaging studies of chondron deformation, as well as zone‐specific finite‐element models of cell‐matrix interactions in cartilage, reveal a biomechanical role for the PCM in cartilage by regulating local stress strain, and fluid flow environments. Furthermore, the PCM may act as a nonlinear mechanical adaptor and protect chondrocytes when a large amount of local strain propagates down to the superficial zone, but it can amplify a lower level of local strain from the middle to the deep zone, providing a mechanical stress gradient reduction environment for chondrocytes (Figure 2).^16^

Once abnormal mechanical stress is applied to the PCM, its degradation is typically observed, causing its layers to thin. The reduction in compressive force is thus altered, preventing generation of an optimal minimal force on the cellular surface. On the other hand, PCM deformation alters the fluid flow in the microenvironment. Therefore, changes in that environment impact the primary cilium and other cell membrane sensors.

### 3.3 Molecules located in the PCM

The PCM contains a set of growth factors and stores regulatory molecules. These molecules bind to specific PCM sites, and most of them are released to manage their concentrations within the PCM, and to respond to mechanical signals. Fibroblast growth factor 2 (FGF‐2) binds to and co‐localizes with HS perlecan within the PCM.^17^ In the resting state, FGF‐2 is thought to be held back from the cell surface, preventing it from transducing signals through its receptor. However, upon stress loading, matrix deformation presents HS‐bound FGF‐2 to its receptor on the cell membrane, resulting in signal transduction by activation of the extracellular signal‐regulated kinase (ERK).^18^ In addition, connective tissue growth factor (CTGF), bone morphogenetic proteins (BMPs), transforming growth factor‐beta (TGF‐β) superfamily members, hepatocyte growth factors (HGFs) and insulin‐like growth factors (IGFs) are known to bind to HS.^19^, ^20^, ^21^ Moreover, Wnt3a binds to biglycan and participates in Wnt signalling through the low‐density lipoprotein receptor 6 (LRP6) receptor, but the mechanisms that initiate the actions of these molecules remain unknown.

## 4 MECHANOSENSORS OF THE CELLULAR MEMBRANE

Articular chondrocytes occupy a dynamic mechanical environment facing a complex and regular combination of shear stress, compression, osmotic stress, hydrostatic pressure and tensile stretch‐induced stress. Mechanical loading within these joints causes chondrocyte deformation and changes in cellular shape and volume. To recognize and respond to mechanical stimulation, chondrocytes use a series of mechanoreceptors to convert extracellular mechanical stimuli from the ECM and the PCM into intracellular biochemical signals. In this section, we discuss the main types of cellular membrane mechanosensors involved in chondrocyte mechanotransduction, including mechanosensitive ion channels (especially Ca^2+^ channels) and the primary cilia and integrin receptors.

### 4.1 Primary cilium

The primary cilium is a cytoskeletal organelle that projects from the cell surface into the extracellular environment. It has been repeatedly demonstrated to be a critical mechanotransducer in several cell types. Chondrocyte primary cilia are sensitive to mechanical changes, and their length decreases significantly upon exposure to hyperosmotic challenges or cyclic tensile strain. In normal cartilage, primary cilial length is 1.1 µm in the superficial layer and 1.5 µm in the deep layer. Primary cilia form structure of microtubules, and their main biological activity is intraflagellar transport (IFT), the process by which soluble proteins and transmembrane receptors move along microtubules. There are two IFT complexes (A and B). IFT‐A binds to the dynein‐2 motor and moves from the cilium tip to the cell body. IFT‐B binds to the kinesin‐II motor and contributes to anterograde transport. There are two subunits of IFT: IFT80, involved in the peripheral subunit of IFT‐B, and IFT 88, the core subunit protein of IFT‐B that acts as a connector between IFT‐A and IFT‐B. These subunits have been investigated and found to be core contributors to chondrocyte function. Evidence from experiments using in vitro fluid flow showed that deflection of the primary cilium occurred, and generated large membrane strains at the ciliary base site. Therefore, a sufficient primary cilium length is required to generate an efficient strain force.

Accordingly, the primary cilium of chondrocytes is critical for cartilage health and homoeostasis. A mutation in *ITF80* in humans can cause a rare disease named Jeune asphyxiating thoracic dystrophy, which is related to cartilage maturation and involves the impairment of endochondral osteogenesis.^22^ *IFT80*‐depleted mice exhibit abnormally short cilia, which are less efficient in mechanical signalling. Conditional depletion of *IFT80* in one study using *Col2α1Cre; Ift80^fl/fl^* mice resulted in impaired chondrocyte differentiation.^23^ In addition, the primary cilium is involved in mechanical load‐related growth plate chondrocyte development. Moreover, transgenic *Col2α1Cre; Ift88^fl/fl^* mice with chondrocyte primary cilial depletion showed elevated levels of osteoarthritic markers, and OA‐like cartilage exhibiting reduced stiffness and increased thickness was observed.^24^ In deep zones, impairment of the *IFT88* complex gene resulted in abnormal cartilage formation and reduced mechanical retention.^25^ Excessive mechanical signalling transduced by key IFTs of the primary cilium mediates transcriptional activity, inhibiting cell differentiation and proliferation.

### 4.2 Channelome

Chondrocytes require an optimal pressure level to maintain haemostasis and react to PCM deformation. This pressure activates a series of extracellular ion channels. The PCM translates mechanical compressive stress into hydrostatic pressure and alters the membrane streaming potentials by regulating the reflux and inflow of sodium, potassium and calcium. Among the ion channels, Na+, K+‐ATPase is an epithelial sodium channel (ENaC) that helps maintain electrochemical gradients. Human cartilage samples and in vitro chondrocyte cell lines confirm that ENaCs are absent in OA because of changes in the active plasma membrane isozyme types, which cause the alternation of sodium and potassium conditions in the PCM and cytoplasm, triggering cartilage degradation.

Most current channelome studies have mainly focused on calcium channels. Changes in intracellular Ca^2+^ concentrations are among the most fundamental molecular responses to physical stimulation, in addition to hydrostatic pressure, osmotic stress, compression and electric currents, which can all be induced by PCM deformation. The roles of Ca^2+^ channels in chondrocyte mechanotransduction and the mechanism by which mechanical stress induces Ca^2+^ signalling are well studied. Normally during the resting phase, an approximately 20 000‐fold Ca^2+^ concentration gradient exists across the cell membrane. Chondrocytes increase calcium concentrations through two common ways: intake from the extracellular environment and release from the intracellular pool. Both mechanisms are involved in Ca^2+^ flow regulation in chondrocytes under physical stress or stimulation.

Typically, three types of calcium channels regulate Ca^2+^ influx: mechanosensitive ion channels (PIEZO channels), transient receptor potential vanilloid channels (TRPV channels) and voltage‐gated calcium channels (T‐type VGCCs).

PIEZO channels are well known as ion channels that are directly activated by mechanical stress. More recently, PIEZO1 and PIEZO2 have been identified as transduction channels that respond to high levels of mechanical stress.^26^ Ca^2+^ influx can be inhibited by blocking PIEZO channels with GsMTx4, and these channels can be activated by high strain, especially in situations of overpressure load or pathological stimulation. Studies have confirmed that PIEZO1 and 2 react under strains of 13%‐45%.^27^, ^28^ Therefore, elevated mechanical stress can produce intracellular Ca^2+^ concentrations that are toxic and can hyperactivate downstream pathways, inducing apoptosis and chondrocyte degradation. However, because PIEZO channels regulate the mechanotransduction pathway, their inhibition protects chondrocytes from overload injuries by reducing cell death rates.^26^

Transient receptor potential vanilloid non‐selective ion channels are Ca^2+^‐preferred channels that are highly expressed in chondrocytes, with significant roles in maintenance of joint health and function.^29^ They mainly respond to physiological mechanical stimulation, as confirmed by in vitro experiments with loading strains of 3%‐8%. As a result, they greatly contribute to maintaining normal intracellular Ca^2+^ concentrations and chondrocyte physiological functions. Accordingly, loss of TRPV channels can disrupt the development and haemostasis of cartilage and induce OA. Blocking TRPV4 channels in vitro reduces chondrocyte sensitivity to osmotic stress and modifies intracellular Ca^2+^ levels.^30^ A previous study of mice with a global *TRPV4* knockout showed that reduced *TRPV4* expression increased age‐related and obesity‐induced OA susceptibility.^31^ Blocking TRPV4 ion channels inhibited production of PCM macro‐components in response to compressive mechanical strain. However, in a normal stress environment the administration of a TRPV4 agonist can stimulate matrix production.^32^ This study revealed that TRPV4 channel activation is a critical mechanism of physical signal transduction and matrix homoeostasis in response to dynamic compressive stress.^32^ Most recently, a study confirmed that excessive mechanical stress induces chondrocyte apoptosis via *TRPV4*.^33^ Additionally, several studies have demonstrated *TRPV5*'s essential function, and its up‐regulation has been observed in a mechanical stress‐related OA rat model. Elevated *TRPV5* increased intracellular Ca^2+^ concentrations and induced chondrocyte apoptosis via the CaMKII‐MAPK and Akt/mTOR pathways.^34^, ^35^

Different levels of physical strain loading involve various chondrocyte mechanotransduction mechanisms. TRPV4 channels mediate physiological signalling, and either gain or loss of function would induce anabolic apoptotic processes in chondrocytes. Injury stresses are converted into cellular transcriptional changes by PIEZO1 and PIEZO2.^28^, ^29^, ^30^, ^31^, ^32^, ^33^, ^34^, ^35^, ^36^ In addition, TRPV4 and PIEZO channels work together to raise currents by forming cell‐substrate contacts, but only PIEZO1 has been shown to regulate stretch‐activated currents—TRPV4 demonstrated no regulatory role in this type of mechanical strain in a whole‐cell patch‐clamp experiment.^37^ In addition to PIEZO and TRPV channels, VGCC voltage‐gated channels also contribute to extracellular Ca^2+^ influx and transduce capacitive‐coupled electrical signals. VGCCs are necessary for PCM component synthesis and chondrocyte electrical stimulation, and inhibition of chondrocyte T‐type VGCCs reduces Ca^2+^ responsiveness by ~50%.^38^ Moreover, an α2δ ligand of the voltage‐gated channel PD‐0200347 mediates cartilage degradation during OA development via Ras‐related inhibition of ERK1/2.^39^

On the other hand, another mechanism has been identified that controls the release of intracellular calcium from the endoplasmic reticulum (ER). Ca^2+^ influx mediated by PIEZO and TRPV channels triggers the release of adenosine triphosphate (ATP). The rate of ATP release increases by almost 10 times under compressive loading (15 kPa). Accumulating ATP can activate purinergic receptors and stimulate production of intracellular phospholipase C to generate inositol 1, 4, 5‐trisphosphate (IP3), which can bind to its receptor on the ER and induce Ca^2+^ release.^40^

The connexin 43 hemichannel located in the primary cilium has been shown to mediate ATP, Ca^2+^ and other small particle exchange between temporomandibular joint (TMJ) chondrocytes and the PCM when chondrocytes are mechanically stimulated.^41^ When mechanical loading opens chondrocyte hemichannels (such as connexin 43), ATP is released, activating P2 receptors via a purinergic pathway, which then induces extracellular and intracellular Ca^2+^ interchange and promotes cellular adaptation to biomechanically changed environments.^42^, ^43^ The synthesis of proteoglycan can be compressively stimulated through a purinergic pathway involving the up‐regulation of cell proliferation and the inhibition of nitric oxide release.^44^

### 4.3 Integrins

Integrins translocate through the cell membrane and are composed of α and β subunits. Integrins help cells attach to the PCM, linking the intracellular cytoskeleton to the extracellular PCM. Integrin‐mediated transduction of extracellular mechanical stimuli as intracellular biochemical signals is dependent upon integrin‐matrix interactions. Within the integrin family, integrin α1β1 has been identified as a key participant in transducing hypo‐osmotic stress,^45^ and the β1 integrin family mainly serves to bind collagens and fibronectin of the PCM. One study showed that integrin α5β1 was responsive to the mechanical stimulation transduction process in regulating cellular polarization.^46^ In another study, it was implicated in the initiation of the mechanotransduction pulse of mandibular condylar chondrocytes (MCCs) under mechanical strain.^47^

Physiologically, integrins mainly support chondrocyte adhesion to the PCM and help maintain its regular shape, which is essential for proliferation. Mice with α1 knockout exhibited fractures due to defective chondrocyte proliferation and a reduced number of mesenchymal progenitors, while mice with a null mutation in the β1 integrin subunit demonstrated a fatal phenotype at a very early embryonic stage. *Col2α1Cre; β1^fl/fl^* mice developed chondrodysplasia due to defective chondrocyte proliferation and migration. A knockout generated deficiency in the major fibronectin binding integrin (α5β1) resulted in early embryonic mortality. In addition, chondrocytes derived from cartilage of patients with OA exhibited membrane depolarization, and their uniformity was quite different from normal healthy articular cartilage because of misconnections between integrins and PCM.^48^

In addition, integrins respond to inflammatory activation in chondrocytes under abnormal mechanical stress. In cartilage tissues from patients with OA, high levels of α1β1 and α3β1 were reported, potentially helping contribute to regulation of PCM deformation and promoting chondrocyte hypertrophy.^49^ Dysregulation of integrins αVβ3 and αVβ5 has been shown to promote joint inflammation and lead to cartilage breakdown and OA, and inhibition of these integrins reduced the expression of interleukin‐1β (IL‐1β) and tumour necrosis factor‐α (TNF‐α).^50^, ^51^ However, IL‐4 demonstrated positive feedback on the expression of integrin β1, while IL‐6 was reduced after the activation of integrins under moderate tensile strain.^52^, ^53^

## 5 PATHWAYS REGULATED BY MECHANOSENSORS

### 5.1 Indian hedgehog pathway

Mechanical stress up‐regulates Indian hedgehog expression (*IHH*) and activates hedgehog (Hh) signalling, but disruption of primary cilial structure by chloral hydrate reverses these changes, indicating that they participate in mechanical transduction signalling.^54^ Cyclic tensile strain activates Hh signalling and promotes the expression of *ADAMTS‐5* in a primary cilia‐dependent manner, but in a high strain environment, histone deacetylase 6 (HDAC6) causes cilial disassembly and blocks this response.^55^, ^56^ A recent in vitro and in vivo study showed that elongation of primary cilia and inhibition of Hh signalling could be produced by chondrocyte treatment with lithium chloride. This suggests that the structure of primary cilia can be modulated by pharmaceutical treatments to regulate the Hh pathway, and such treatments could be useful for OA therapy.^57^

### 5.2 Wnt pathway

Wnt signalling participates in chondrocyte formation during foetal development via β‐catenin activity. A study demonstrated that increased expression of genes involved in Wnt signalling impairs chondrogenesis in vivo and in vitro, and reduces PCM synthesis.^58^ Evidence has shown that some core Wnt pathway components localize to primary cilia via IFT binding.^59^ IFT80 silencing in bone marrow–derived stromal cells disrupts chondrogenic differentiation and causes Wnt activation.^60^ IFT80 knockout also disrupts primary cilial formation, alters Wnt signalling and ultimately blocks chondrocyte differentiation. *Col2α*Cre; *Ift88^fl/fl^* mice show aberrant transduction of Wnt and Hh signalling in the growth plate, suggesting that both ITF80 and ITF88 are main effectors of primary cilia in response to mechanical signalling, and have critical roles in Wnt pathway regulation and chondrogenesis.^61^, ^62^

### 5.3 MAPK‐ERK pathway

Various clinical studies have found that the mitogen‐activated protein kinase/extracellular signal‐regulated kinase (MAPK‐ERK) pathway is critical for chondrocyte differentiation and haemostasis, and that its inhibition impairs early chondrocyte differentiation and hypertrophic chondrocyte differentiation.^63^ Moreover, it is involved in mechanotransduction, regulated by mechanosensors on the cellular membrane.

Calcium channels, primary cilia, TRPV4 and p38 all contribute to ERK activation, and the latter two also participate in early responses to hypo‐osmotic stress and elevated intracellular calcium.^64^ Most recently, a study demonstrated that Cbp/p300‐interacting transactivator 2 (CITED2) transactivation is essential for strain‐induced signal generation. This process is mediated through primary cilia and involves ATP exportation, Ca^2+^ transduction and ERK1/2 phosphorylation. Induction of *CITED2* had an anticatabolic effect by reducing the volume of matrix metalloproteinases 1 and 13 (MMP‐1 and MMP‐13) and initiating autophagy.^65^, ^66^

In addition, integrins are shown to interact with the MAPK‐ERK pathway. One study demonstrated that calcium‐ and integrin‐associated Src kinases converge to activate the ERK‐MAPK‐p38 pathway after mechanical stimulation, and calcium assists in the regulation of the integrin‐mediated signalling pathway.^67^ A previous study demonstrated that mechanical stimulation of approximately 0.33 Hz induces tyrosine phosphorylation of the pp125FAK protein kinase.^68^ Periodic mechanical stresses in the range of 0‐200 kPa and 0.1 Hz promote rat matrix synthesis and chondrocyte proliferation via integrin β1‐ERK1/2 signals, including integrin β1‐Src‐PLCγ1/Rac1‐ERK1/2, integrin β1‐CaMKII‐Pyk2‐ERK1/2, integrin β1‐Src‐Rac1‐FAK(Tyr576/577)‐ERK1/2 and integrin β1‐FAK (Tyr397)‐ERK1/2.^69^ Most recently, differentially expressed genes were identified using quantitative proteomic analysis in chondrocytes under pressure. Growth factor receptor‐bound protein 2 (GRB2) increased 1.49‐fold in the dynamic stress group, which was mediated through integrin β1 and led to increased phosphorylation of FAK and ERK1/2.^70^ Therefore, the MAPK‐ERK pathway is thought to be a downstream target of several mechanosensors.

### 5.4 TGF‐β1 pathway

TGF‐β1 signalling is commonly activated during mechanical stress transduction, but its precise role has been debated for decades and a series of studies have attempted to address how it induces OA.^71^, ^72^ TGF‐β1 is a secreted protein that binds to its cell surface receptor (especially TGF‐β type I (TGFBR1) and TGF‐β type II (TGFBR2)), initiating diverse cellular responses. Subsequently, the activated receptor targets downstream molecules, directly phosphorylates SMAD2 and 3, and forms a heterotrimeric complex by activating SMAD2/3 with SMAD4.^73^, ^74^, ^75^ This complex translocates into the nucleus and targets other regulatory factors, activating or inhibiting gene expression.^76^ In the resting stage, TGF‐β1 is located in the PCM, but when abnormal mechanical stress is applied, some of it is released to target its receptor.

Immature mice with truncated *TGFBR2* developed OA‐like joints and increased collagen type X at 4 weeks of age.^77^ In addition, *TGFBR2* deletion in type II collagen‐expressing cells of *Tgfbr2Col2ER* mice also resulted in an OA phenotype as early as 2 weeks of age.^78^ Knockout of downstream *SMAD3* also induced early‐onset OA, with enhanced chondrocyte hypertrophy. Therefore, TGF‐β1 signalling is essential for developmental cartilage formation because it suppresses *RUNX2* and *MMP‐13* expression, which contribute to degeneration of articular cartilage.^79^

However, in mature chondrocytes, TGF‐β1 overexpression induces osteophyte formation and PG over‐recruitment, an early sign of articular cartilage degradation.^72^ Additionally, elevated TGF‐β1 and p‐SMAD2/3 were found in abnormal mechanical stress‐induced OA models. Elevated expression of p‐SMAD2/3 was also observed in addition to an increase in the high‐temperature requirement A1 protease (HtrA1) in knee joints.^80^ These results suggest that HtrA1 is also induced by activated canonical TGF‐β1 signalling in mouse OA models. HtrA1 is also involved in Toll‐like receptor signalling and Wnt/β‐catenin signalling, and it induces PCM degeneration and promotes the association of discoidin domain receptor 2 (DDR2) with type II collagen.^81^, ^82^ Activated DDR2 up‐regulates the expression of *MMP‐13* through ERK and p38, accelerating degradation of type II collagen and PGs.^83^ Based on TGF‐β1's biological signalling functions, *AgcCreERT2; Tgfbr2^fl/fl^* mice were used to produce a tissue‐specific TGFBR2 deletion in mature chondrocytes in a partial discectomy (PDE)‐induced mechanical stress‐related TMJOA model, revealing delayed cartilage degradation.^20^ Similar results have been reported using *Ddr2^±^* and *Ddr2^−/−^* mice.^81^

### 5.5 Hippo‐YAP pathway

**FIGURE 3:**
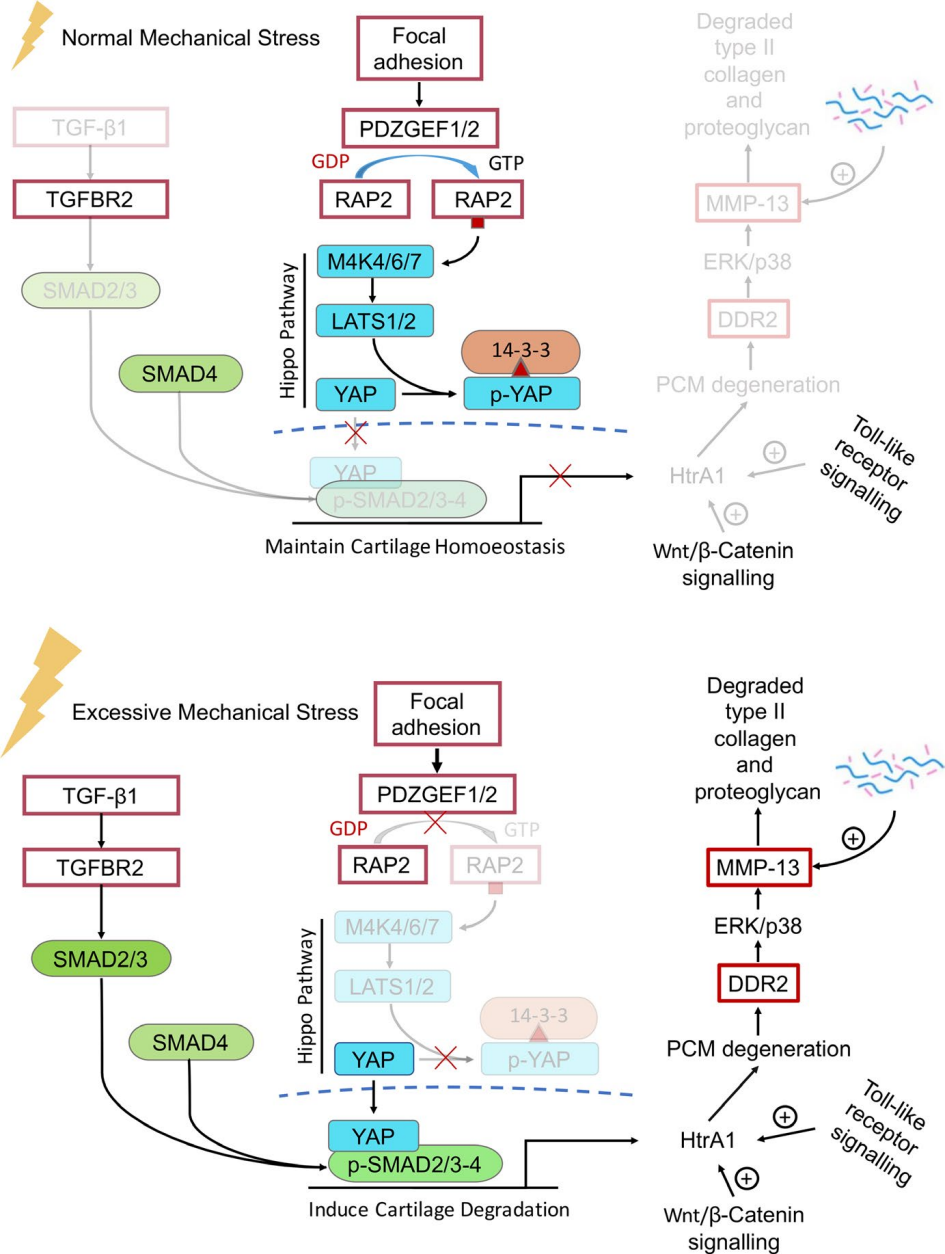
The interaction between Rap2‐Hippo‐Yap1 pathway and TGF‐β signaling

YAP and TAZ are fundamental effectors in mechanical stress transduction and the regulation of cell proliferation and differentiation, which are phosphorylated downstream of the Hippo pathway. Under normal mechanical stress, activated Hippo pathway proteins (including MAP4K4, MAP4K7, ARHGAP29, LATS1/2 and MOB1/2) phosphorylate YAP/TAZ, confining them to the cytoplasm. Under excessive mechanical stress, Hippo is deactivated, losing the ability to control YAP/TAZ, which translocate into the nucleus as cotranscription factors that interact with the SMAD and TEAD family to develop transcript complexes targeting *HTRA1*, *CTGF*, *CYR61* and *SOX9*. According to stem cell studies, when cells are shifted from stiff to soft culture matrices, YAP and TAZ translocate from the nucleus to the cytoplasm and are then inactivated. Therefore, they are extremely sensitive to changes in mechanical stress, and as several studies have demonstrated, there is a connection between them and TGF‐β1 signalling. TGF‐β1 signalling is well understood as a mechanotransducer that induces OA by activating p‐SMAD2/3 and then targeting the *HTRA1*‐DDR2‐*MMP‐13* pathway. Although limited studies have documented YAP/TAZ regulatory functions relating to this pathway, the interaction between YAP/TAZ and p‐SMAD2/3 is well known to form a complex after YAP/TAZ translocates into the nucleus to regulate downstream gene expression.^84^ Activated YAP/TAZ is essential for TGF‐β1 signalling and induces the *HTRA1‐*DDR2‐*MMP‐13* process, leading to OA pathology (Figure 3).^84^

YAP inactivation is required for the maintenance of chondrogenic phenotypes, and a connection between this inactivation and MSC/chondrocyte fates has been demonstrated in a fluid flow‐induced mechanical microenvironment.^85^, ^86^ Another research group revealed that YAP was also a negative regulator of chondrogenesis in mesenchymal stem cells.^87^ Based on these results, YAP/TAZ has been proven essential effectors that can transduce mechanical stress into the nucleus and help maintain normal chondrocyte development, maturation and homoeostasis. On the other hand, elevated *YAP1* expression has been observed in OA cartilage tissue, and overexpressed *YAP1 i*nteracts with Beclin‐1 and promotes OA progression. Researchers have attempted to use siRNA to inhibit *YAP1* in a mechano‐induced OA mouse model, and they found that its inhibition prevented cartilage degradation and ameliorated OA development.^88^, ^89^

Rap2, a Ras‐related GTPase, was recently identified as a key intracellular signal transducer that controls mechanosensitive cellular activities through YAP and TAZ.^90^ Mechanistically, matrix stiffness influences the levels of phosphatidylinositol 4,5‐bisphosphate and phosphatidic acid via phospholipase Cγ1 (PLCγ1), leading to Rap2 activation through PDZGEF1 and PDZGEF2. Therefore, Rap2 is a pioneer that converts mechanical signals from physical stress to biochemical molecular activation. Deletion of *RAP2* increased nuclear localization of YAP/TAZ, but its functions in chondrogenesis and OA development are unknown. However, as the connection between *RAP2* and YAP/TAZ is well established, we believe more research should focus on its role in OA.

## 6 miRNA RESPONSES TO MECHANICAL SIGNALLING

MicroRNAs (miRs) are a class of small conserved single‐stranded non‐coding RNAs that regulate gene expression by degrading target mRNAs or inhibiting their translation. MiRs function as post‐transcriptional regulators in a stimulus‐dependent manner. A series of studies have investigated their role in cartilage development and the initiation of OA.^91^ Although miRs are important modulators of epigenetic regulation, there is little evidence that they affect mechanical signalling responses by silencing target mRNAs.^92^ The first described mechanosensitive miR was miR‐365, which modulates chondrocyte proliferation and hypertrophy by targeting *HDAC4*.^93^ MicroRNAs 221/222 were next identified and displayed increased expression in weight‐bearing joint cartilage compared with non‐weight‐bearing cartilage. Several studies suggest that levels of miR‐27a, miR‐140 and miR‐146a are altered when monolayer cultures of human chondrocytes are exposed to hydrostatic pressure, but study limitations prevented definitive conclusions. Most recently, the profile of mechanosensitive miRs regulated by anabolic and catabolic cartilage loading was studied. In that study, miR‐221, miR‐6872‐3p and miR‐6723‐5p were up‐regulated under both anabolic and catabolic loading and were validated, while others (miR‐199b‐5p, miR‐1229‐5p, miR‐1275, miR‐4459, miR‐6891‐5p and miR‐7150) only showed changes after catabolic loading.^91^ In addition, miR‐221 is regulated by ERK1/2 signalling and is only induced by mechanical loading following stimulation by one of the most prominent mechanotransduction pathways: the MAPK‐ERK1/2 pathway.^94^ Additionally, miR‐221 recognizes a 3′UTR of α10‐integrin mRNA, but other miRs have not demonstrated such clear functional evidence.

## 7 EXPERIMENTAL APPLICATION OF MECHANICAL STRESS TO CHONDROCYTES

Chondrocytes are always subjected to physiological mechanical stimulation in joints, but a set of pathological phenotypes appears to result from abnormal mechanical stress. Therefore, several approaches have been developed to analyse the impacts of mechanical stress on chondrocytes, which have attempted to model actual physiological conditions.

Therefore, the best method is to test the cell in vivo. A number of surgical approaches have been described to apply excess mechanical stress to the knee joint and TMJ. For the knee joint, anterior cruciate ligament transection (ACLT)^95^ and destabilization of the medial meniscus (DMM)^96^ surgeries have been applied to induce unstable knee joints and artificially generate an overloading of mechanical stress. In addition, a surgical approach similar to PDE has been developed to remove part of the joint disc, resulting in locally elevated mechanical stress.^20^ Moreover, an orthodontic method was invented to generate unilateral anterior crossbite (UAC), which produces excessive mechanical stress on TMJ chondrocytes and demonstrates a practical way to analyse how mechanotransduction occurs in TMJ, and its role in TMJOA.^97^

Although in vivo studies are the most practical for analysing pathophysiological processes, some complicated experiments still need to be completed in vitro, requiring methods to establish a suitable environment for subjecting cultured chondrocytes to mechanical stresses. 3D culture systems have been used to solve this issue, including both scaffolded and scaffold‐free approaches. A series of synthetic materials have been used to build these scaffolds, including calcium phosphate, poly lactic‐co‐glycolic acid (PLGA), PS, polyalanine‐poloxamer‐polyalanine and collagen type I‐glycosaminoglycan.^98^, ^99^, ^100^ A scaffold‐free method using a gel for cell seeding is able to receive extra‐mechanical stress interventions. Examples of this approach include alginate (Alg)/hyaluronic acid (HA) and Alg/hydroxyapatite (Hap) gels.^101^ Additionally, a hydrogel has been applied to establish a 3D structure for chondrocyte culture.^102^

In addition, some 2D monolayer methods still offer great utility for testing different kinds of mechanical stress in chondrocytes. A method using fibronectin‐coated acrylamide hydrogels to generate varying stiffnesses (between 0.7 and 40 kPa) for cultured cells has been applied for several years and been widely used to improve basic understanding of the biological particles involved in mechanotransduction.^103^ In addition, some types of equipment, such as Flexcell® devices, have been used to culture monolayers of chondrocytes and take advantage of material shaping to generate tensile or shear mechanical stresses.^53^

## 8 TARGETING MECHANOTRANSDUCTION PATHWAYS FOR OA THERAPY

Excessive mechanical stress induces OA via the mechanotransduction pathway, suggesting that blocking it might help to treat osteoarthritis, and several methods have been developed to help attain this medical goal.

In addition, small molecular inhibitors (SMIs) are optimal choices for mechanotransduction pathway targets. Halofuginone is a synthetic halogenated derivative of febrifugine, a natural quinazolinone alkaloid derived from the Chinese herb *Dichroa febrifuga*. It has been shown to inhibit TGF‐β1 signalling activation. Halofuginone has been applied in an anterior cruciate ligament transection (ACLT) mouse model of OA, and OA progression was shown to be suppressed through inhibition of TGF‐β1/SMAD2/3 signalling.^104^ Another SMI, WRG‐28, specifically targets the DDR2 extracellular domain and inhibits receptor signalling, providing opportunities to develop new highly selective drugs to block mechanotransduction pathways.^105^ Other research has used a specific TGF‐β1 inhibitor to protect joints in a rat model.^106^

Non‐coding RNAs, especially miRs, have been studied thoroughly, and techniques have been developed to produce modified oligonucleotides that inhibit specific miRNAs. For example, locked nucleic acid miR‐181a‐5p antisense oligonucleotides (ASOs) reduced the expression of miR‐181a‐5p in lumbar facet joint cartilage and knee joints demonstrating the protective effects of miR treatments on cartilage degradation.^107^ In previous studies, a series of miRs has been implicated in mechanotransduction pathways, and the ASO technique could provide new ways to use miRs to treat OA.

## 9 CONCLUSION

Chondrocytes are mechanosensitive, requiring regular stimuli for optimal functioning. Research has focused on their mechanical environment and mechanotransduction mechanisms. Experimental PCM studies and theoretical models of cell‐matrix interactions indicate that distinct properties of the PCM can regulate chondrocyte mechanical environments, influencing cartilage homoeostasis and joint health. Individual PCM components and growth factors may serve as sensors and effectors of mechanical signals. Insights into the PCM structure, composition and mechanical properties under normal and diseased conditions may expand our understanding of chondrocyte mechanotransduction. Changes in interstitial fluid, hydrostatic pressure, osmotic stress and streaming potentials are sensed by chondrocytes through mechanoreceptors in their plasma membranes. These receptors consist of ion channels, primary cilia and integrin receptors, and they transduce extracellular physical stimulation and cytoskeletal complex signals that are responsible for intracellular communication. Further study of these pathways in chondrocytes may provide new insight into osteoarthritis pathogenesis, and new therapeutic approaches for this disease.

## CONFLICT OF INTEREST

The authors report no conflict of interest. The authors alone are responsible for the content and writing of the paper.

## AUTHORS' CONTRIBUTIONS

Jie Fang and Zhihe Zhao generated the concept of the review. Zhenxing Zhao, Mengjiao Wang and Sen Zhao participated in articles review and data extraction. Yifei Li draw the figures for this review. Zhenxing Zhao and Yifei Li wrote the manuscript. Finally, Zhihe Zhao and Jie Fang revised and approved this manuscript.
